# Ancient out-of-Africa migration of *Plasmodium falciparum* along with modern humans

**DOI:** 10.1186/1475-2875-9-S2-O30

**Published:** 2010-10-20

**Authors:** Kazuyuki Tanabe, Toshihiro Mita, Francois Balloux

**Affiliations:** 1 Laboratory of Malariology, Research Institute for Microbial Diseases, Osaka University, Osaka 565-0871, Japan; 2 Department of International Affairs and Tropical Medicine, Tokyo Women's Medical University, Tokyo 162-8666, Japan; 3 MRC Centre for Outbreak Analysis and Modelling, Department of Infectious Disease Epidemiology, Faculty of Medicine, Imperia l College,London W2 1PG, UK

## 

Genetic diversity of *Plasmodium falciparum* is a key issue for better underÂ¬standing of mortality, morbidity and effective control of malaria. However, little is known about the genetic makeup of *P. falciparum* populations.

It is generally assumed that the parasite population structure has been shaped by a variety of factors including transmission intensity, past intervention efforts and evolutionary history. Since *P. falciparum* is a parasite of man, its demographic history is expected to be intimately associated with that of modern humans. To assess the relative importance of human demographic history, we characterised spatial patterns of genetic diversity of *P*. *falciparum* populations by analyzing a worldwide sample of >500 isolates sequenced for two housekeeping genes (*serca* and *adsl:* 63 SNPs from 5.0 kb per isolate). We observed a strong negative correlation between within-population genetic diversity and geographic distance from Africa over Africa, Asia and Oceania, presenting evidence for isolation by distance pattern in *P. falciparum.* This negative correlation was also noted for two surface antigen genes (*msp1* and *csp:* 165 SNPs from 5.5 kb per isolate).

In contrast, regional variation in transmission intensity and recent control initiatives seem to have had a negligible impact on the distribution of genetic diversity, indicating that geography is the primary predictor of the genetic diversity of *P. falciparum* populations. The striking geographic patterns of isolation by distance mirror the ones previously documented in modern humans. Age estimates of *P. falciparum* populations, based on genetic distance between *P. falciparum* and *P. reichenowi,* a chimpanzee malaria parasite, strongly suggest that modern humans were infected prior to their exit out-of-Africa and carried the parasite along during their colonization of the world.
